# Phase I study of adjuvant chemotherapy with gemcitabine plus cisplatin in patients with biliary tract cancer undergoing curative resection without major hepatectomy (KHBO1004)

**DOI:** 10.1007/s00280-014-2431-y

**Published:** 2014-03-11

**Authors:** Masanori Toyoda, Tetsuo Ajiki, Yutaka Fujiwara, Hiroaki Nagano, Shogo Kobayashi, Daisuke Sakai, Etsuro Hatano, Masashi Kanai, Shoji Nakamori, Atsushi Miyamoto, Akihito Tsuji, Satoshi Kaihara, Hisashi Ikoma, Shigekazu Takemura, Hideyoshi Toyokawa, Hiroaki Terajima, Satoshi Morita, Tatsuya Ioka

**Affiliations:** 1 Department of Medical Oncology and Hematology, Kobe University Hospital, 7-5-2, Kusunoki-cho, Chuo-ku, Kobe, 650-0017 Japan; 2 Division of Hepato-Biliary-Pancreatic Surgery, Department of Surgery, Graduate School of Medicine, Kobe University, Kobe, Japan; 3 Department of Experimental Therapeutics, Exploratory Oncology Research and Clinical Trial Center, National Cancer Center Hospital, Tokyo, Japan; 4 Department of Gastroenterological Surgery, Graduate School of Medicine, Osaka University, Osaka, Japan; 5 Department of Frontier Science for Cancer and Chemotherapy, Graduate School of Medicine, Osaka University, Suita, Japan; 6 Department of Surgery, Graduate School of Medicine, Kyoto University, Kyoto, Japan; 7 Department of Clinical Oncology and Pharmacogenomics, Graduate School of Medicine, Kyoto University, Kyoto, Japan; 8 Department of Surgery, National Hospital Organization Osaka National Hospital, Osaka, Japan; 9 Department of Medical Oncology, Kobe City Medical Center General Hospital, Kobe, Japan; 10 Department of Surgery, Kobe City Medical Center General Hospital, Kobe, Japan; 11 Division of Digestive Surgery, Department of Surgery, University Hospital, Kyoto Prefectural University of Medicine, Kyoto, Japan; 12 Department of Hepatobiliary Pancreatic Surgery, Graduate School of Medicine, Osaka City University, Osaka, Japan; 13 Department of Surgery, Kansai Medical University, Hirakata, Japan; 14 Department of Gastroenterological Surgery and Oncology, Kitano Hospital, The Tazuke Kofukai Medical Research Institute, Osaka, Japan; 15 Institute for Advancement of Clinical and Translational Science, Kyoto University Hospital, Kyoto, Japan; 16 Hepatobiliary and Pancreatic Oncology, Osaka Medical Center for Cancer and Cardiovascular Diseases, Osaka, Japan

**Keywords:** Gemcitabine and cisplatin, Adjuvant chemotherapy, Biliary tract cancer, Phase I

## Abstract

**Purpose:**

We conducted a phase I study to determine the maximum tolerated dose and recommended dose (RD) of this gemcitabine plus cisplatin (GC) combination in the adjuvant setting for biliary tract cancer (BTC). GC has become a standard chemotherapy regimen for patients with locally advanced or metastatic BTC; however, the benefit of adjuvant therapy for BTC is unclear.

**Methods:**

Patients with BTC were eligible if they met the following criteria: Stage IB or higher; and undergoing resection without major hepatectomy. The starting dose matched the standard dose of gemcitabine (1,000 mg/m^2^) and cisplatin (25 mg/m^2^) on days 1 and 8, every 3 weeks for up to 24 weeks. The dose limiting toxicities (DLTs) were determined during the first 6 weeks, and a 3+3 dose finding design with cohorts of 3–6 patients was used. Further cohort expansion took place.

**Results:**

One DLT, namely grade 4 neutropenia, was observed among six patients at the starting dosages. Then, we expanded the cohort with a total of eighteen patients to evaluate RD and no further DLTs were observed. During the entire study, the most common grade 3/4 adverse events were neutropenia (94 %) and leucopenia (56 %). Non-hematological toxicities were manageable.

**Conclusions:**

We defined the standard dose of GC as the RD for adjuvant chemotherapy for BTC treated by curative resection without major hepatectomy. Further study is warranted to clarify the safety and efficacy of this regimen for all patients.

## Introduction

Biliary tract cancer (BTC) includes intrahepatic cholangiocarcinoma, extrahepatic cholangiocarcinoma, gallbladder cancer and ampullary cancer. Although the incidence rates of BTC are relatively low in the USA and some western European countries, the rates are high in Asia, Latin America and eastern European countries [1]. There were almost 18,000 deaths in Japan and 3,200 deaths in the USA from extra-hepatic bile duct cancer, ampullary cancer and gallbladder cancer [2, 3]. The prognosis of BTC patients is extremely poor, with five-year survival rates in the range of 5–15 % [4, 5]. Even though surgical resection is the only curative treatment, the reported overall survival rate following surgery is nevertheless only about 40 % [6]. To improve the prognosis of BTC requires the development of effective adjuvant or neoadjuvant treatment with multidisciplinary care.

To date, only two randomized control trials (RCTs) of adjuvant chemotherapy for BTC have been reported, but both included patients with BTC in limited locations. These studies showed no significant benefit from adjuvant chemotherapy. Takada et al. [7] reported the results of postoperative adjuvant chemotherapy in patients with pancreatic cancer and limited BTC, but an intention-to-treat analysis showed no significant difference in five-year survival. The ESPAC-3 trial was a multicenter RCT of adjuvant chemotherapy versus observation with periampullary adenocarcinoma. Although multivariable analysis with adjustment for prognostic variables demonstrated a statistically significant survival benefit associated with adjuvant chemotherapy, median survival time from the primary analysis in the chemotherapy group did not significantly differ from that in the observation group [8].

In another study, a meta-analysis found a non-significant improvement in overall survival with adjuvant therapy (chemotherapy, radiotherapy or chemoradiotherapy) compared with surgery alone for BTC, but patients with node-positive and R1 disease had a significant survival benefit [9]. The authors accordingly reported that adjuvant therapy is the standard of care for high-risk patients. However, this meta-analysis had limitations, included selection bias, stage migration and heterogeneous treatments and should not be considered conclusive, thereby warranting additional prospective studies [10]. Thus, an effective evidence-based adjuvant chemotherapy has yet to be identified, as reflected by guidelines of the National Comprehensive Cancer Network [11] and from guidelines in Japan [12] which strongly endorse the need for clinical trials of adjuvant chemotherapy for patients with resectable BTC.

At this point, however, a standard adjuvant chemotherapy for BTC has not been determined. It is necessary to develop more effective adjuvant chemotherapy to suppress recurrences after surgery. We speculated that combination therapy with gemcitabine plus cisplatin (GC) would be an effective and intensive adjuvant chemotherapy, because in patients with locally advanced or metastatic BTC, the ABC-02 trial showed a significant survival advantage for GC over gemcitabine alone [13], and similar results were observed in Japan [14]. Therefore, we conducted a phase I study to determine the maximum tolerated dose (MTD) and recommended dose (RD) of GC in patients with BTC undergoing curative resection without major hepatectomy.

## Patients and methods

### Eligibility criteria

Patients with BTC who met the following criteria were eligible: histologically confirmed BTC (intrahepatic or extrahepatic cholangiocarcinoma, gallbladder cancer or ampullary cancer); Stage IB disease or higher; complete macroscopic (R0 or R1) resection without major hepatectomy (resection of more than three segments defined according to Couinaud’s classification); age over 20 years; Eastern Cooperative Oncology Group performance status of 0–1; adequate marrow function (neutrophil count ≥1,500/mm^3^, platelet count ≥100,000/mm^3^); adequate liver function [total serum bilirubin ≤3 times the upper limit of normal (ULN); aspartate aminotransferase and alanine aminotransferase levels ≤5 times the ULN]; adequate renal function (serum creatinine concentration ≤1.2 mg/dl and creatinine clearance ≥50 ml/min); adequate oral intake; and written informed consent. Patients satisfying these criteria started the regimen within 4–12 weeks after surgical resection. Exclusion criteria were the presence of pulmonary fibrosis or interstitial pneumonia, severe heart disease, uncontrolled diabetes mellitus, and active infection; pregnancy or lactation; women of childbearing age, unless using effective contraception; severe drug hypersensitivity; mental disorder; concurrent double cancer; and other serious medical conditions. The study protocol was approved by the institutional review board of each participating institution.

### Study design

This phase I study (ClinicalTrials.gov ID NCT01297998; UMIN ID 000004622) was designed by the Kansai Hepatobiliary Oncology Group (KHBO). Patient registration and data management were conducted at an independent data center at the Osaka Medical Center for Cancer and Cardiovascular Diseases. All laboratory tests required to assess eligibility were completed within 14 days before the start of protocol treatment. Planned dosages of gemcitabine (mg/m^2^)/cisplatin (mg/m^2^) were as follows for 6 months. Level 1 (starting level): 1,000/25 on day 1 and 8, every 3 weeks. Level 0: 800/25 on day 1 and 8, every 3 weeks. Level-1: 1,000/25 on day 1, every 2 weeks. Level-2: 800/25 on day 1, every 2 weeks.

### Definition of DLTs, MTD and RD

Dose limiting toxicities (DLTs) were determined during the first 6 weeks. DLT was defined according to Common Toxicity Criteria Adverse Events v4.0 (CTCAE v4.0) as one or more of the following events: (1) grade 4 leucopenia, neutropenia or thrombocytopenia, (2) grade 3–4 neutropenia complicated by fever, (3) clinically significant grade 3–4 non-hematological toxicities, (4) need to withhold GC on day 8 consecutively in the first 6 weeks due to an adverse event or (5) delay in course of more than 14 days following an adverse event. If DLT was observed in one or two of three patients, three additional patients were enrolled at that level. If three or more of six patients or if all three initial patients experienced DLT at that level, the dose was de-escalated to the next lower level. MTD was defined as the lowest dose level that produced DLTs in three or more of six patients or in all three initial patients. RD was defined as one dose level lower than the MTD, considering the toxicity and tolerability observed in the entire study. If a DLT was observed in one or two of six patients in Level 1 (starting dose), Level 1 was initially defined as the RD. The protocol required the enrollment of additional patients to examine the safety of the RD according to the recommendation from the Data and Safety Monitoring Committee (DSMC). There was no dose escalation in individual patients.

### Treatment

Chemotherapy was started and repeated on day 1 if absolute neutrophil count was ≥1,000/mm^3^; platelet count was ≥100,000/mm^3^; total bilirubin was ≤3 times the ULN; AST/ALT was ≤five times the ULN; creatinine was ≤1.2 mg/dl; and any non-hematological toxicities were ≤grade 1. If the patient did not meet the above criteria, chemotherapy was delayed by 1 week or more until recovery. On day 8, chemotherapy was started and repeated if absolute neutrophil count was ≤1,000/mm^3^; platelet count was ≤70,000/mm^3^; creatinine was ≤1.5 mg/dl; and any non-hematological toxicities were ≤grade 1. GC dose was reduced to −1 level if an adverse event defined as a DLT occurred. The protocol allowed a single dose reduction only, and no re-escalation was allowed. Protocol treatment was discontinued in individual patients if any of the following occurred: completion of protocol treatment for 6 months; disease recurrence or death; need for a second dose reduction; delay in schedule of 1 month or more; patient refusal to further treatment; or at the discretion of the attending physician.

### Pretreatment and follow-up evaluation

Pretreatment evaluation included the patient’s medical history and physical examination, imaging tests using contrast-enhanced computed tomography or magnetic resonance imaging, blood tests, electrocardiogram and chest X-rays. Creatinine clearance was calculated using the Cockcroft-Gault formula. During treatment cycles, physical examination and blood tests were scheduled on each administration day of chemotherapy. Carcinoembryonic antigen and carbohydrate antigen 19-9 (CA19-9) were measured at the time patients were enrolled in the study and every month thereafter. Toxicity was evaluated using the CTCAE v4.0. All the safety data during adjuvant therapy, even if it was off protocol, were included for analysis. Imaging tests were planned at every 12 weeks after the start of treatment, and additional imaging tests were performed to confirm recurrence if clinically suspected. Treatment duration day was defined from the administration of GC to end of the protocol treatment.

## Results

### Patient characteristics

From July 2011 to July 2012, 18 patients from nine institutions were enrolled (Table 1). Median age was 70.5 years (range 55–74). Nine patients (50 %) had extrahepatic bile duct cancer, four (22 %) had gallbladder cancer, four (22 %) had ampullary cancer and one (6 %) had intrahepatic bile duct cancer. Pancreatoduodenectomy was performed in 12 patients (67 %), simple to a radical or extended cholecystectomy in 5 (28 %), and bile duct resection and partial hepatectomy in 3 (17 %). Median time of initiation of adjuvant therapy after surgical intervention was 49.7 days (range 29–72). Postoperative complications were seen in six patients (33 %). Fifteen patients (83 %) had a negative margin (R0) and three (17 %) had a microscopically positive margin (R1).

**Table 1:** Baseline characteristics of patients (*N* = 18)

Sex (M/F)	13/5
Age (years) (median, range)	70.5 (55–74)
Primary lesion	
Intrahepatic	1
Extrahepatic	9
Gallbladder	4
Ampulla of vater	4
Resectability (R0/R1)	15/3
Performance status (0/1)	15/3
TNM	
T1/T2/T3/T4	1/7/7/3
N0/N1	41,831
UICC-Stage 7th IB/IIA/IIB/III/IV	4/2/9/2/1
Postoperative complications^a^	
None	13
Pancreatic fistula	4
Wound infection	1
Bleeding	1
Operative procedure^a^	
Pancreatoduodenectomy	12
Bile duct resection	3
Cholecystectomy	5
Partial hepatectomy	3
Time of initiation of adjuvant therapy (days) (median, range)	49.7 (29–72)

^a^Some are overlapping

### DLT, MTD and RD

One DLT was observed in three patients at Level 1 during the first 6 weeks, namely grade 4 neutropenia. This dosage level was then expanded with three additional patients, and no further DLTs were observed. According to the recommendation from the DSMC, a total of 18 patients were enrolled at Level 1 to thoroughly evaluate DLT and RD, then one patient who withdrew consent was excluded from evaluation of DLT. One DLT among 17 patients was observed in Level 1 during the first 6 weeks. Throughout the entire study, six (33 %) patients completed protocol treatment. Eight (44 %) dropped out due to the need for a second dose reduction. Two did not meet starting criteria for creatinine with a grade 1 increase in CTCAE v4.0. One had rapid tumor progression and died within 30 days after the last administration of GC. One withdrew consent.

Overall, we ensured the safety of Level 1, the standard dose of the GC regimen, and defined it as the RD. MTD was not defined in this study because we did not plan to escalate the dosage of GC more than the standard dose.

### Hematological adverse events

Overall, hematological toxic events were common, the most common of which was neutropenia (Table 2). Grade 3–4 neutropenia, leucopenia, anemia and thrombocytopenia were observed in 56, 33, 0 and 6 % of patients during the first 6 weeks and in 94, 56, 22 and 6 % of patients throughout the entire course of treatment, respectively. Febrile neutropenia was not seen in this study.

**Table 2:** Hematological adverse events

*N* = 18	First 6 weeks					Entire course				
Grade (NCI-CTCAE v4.0)	1	2	3	4	Gr 3–4 (%)	1	2	3	4	Gr 3–4 (%)
Neutropenia	1	6	9	1	56	0	1	13	4	94
Leukopenia	4	6	6	0	33	2	6	10	0	56
Anemia	8	5	0	0	0	5	8	4	0	22
Thrombocytopenia	6	3	1	0	6	6	4	0	1	6

*CTCAE* common terminology criteria for adverse events

### Non-hematological adverse events

Table 3 summarizes the non-hematological adverse events reported during the first 6 weeks and throughout the entire course of treatment according to CTCAE v4.0. The major adverse events were anorexia, nausea, constipation and malaise. One patient developed pneumonia with grade 1–2 neutropenia; he was admitted for treatment with an intravenous antibiotic and recovered well within 1 week. Throughout the entire treatment, two patients did not meet the starting criteria for creatinine, with a grade 1 increase in CTCAE v4.0. Few grade 3–4 adverse events were observed, and all non-hematological toxic effects were manageable.

**Table 3:** Non-hematological adverse events

*N* = 18	First 6 weeks					Entire course				
Grade (NCI-CTCAE v4.0)	1	2	3	4	Total (%)	1	2	3	4	Total (%)
Constipation	8	1	0	0	50	8	1	0	0	50
Anorexia	4	4	0	0	44	6	3	1	0	56
Nausea	4	3	0	0	39	5	4	0	0	50
Malaise	4	2	0	0	33	4	3	1	0	44
Vomiting	3	0	0	0	17	2	1	0	0	17
Abd. pain	2	0	0	0	11	2	0	0	0	11
Rash	2	0	0	0	11	3	0	0	0	17
Diarrhea	0	1	0	0	6	1	1	0	0	11
Stomatitis	1	0	0	0	6	4	0	0	0	22
Dysgeusia	1	0	0	0	6	4	0	0	0	22
Alopecia	0	0	0	0	0	3	0	0	0	17

10 % or more frequent adverse events are listed in this table

### Treatment course

Treatment course is shown in Table 4. The planned duration of adjuvant GC was 6 months. Median and mean duration of treatment was 142.5 (range 15–183) and 127 days, respectively. Median number of GC administration was 10 times (range 1–16). Median and mean cumulative doses of gemcitabine were 9,900 (range 1,000–15,000) and 9,289 mg/m^2^, respectively, while those of cisplatin were 250 (range 25–400) and 244 mg/m^2^, respectively. Eleven patients required a dose reduction in GC from Level 1 to Level 0.

**Table 4:** Course of treatment

Patient no.	Dose level	Age (years)	Sex	Number of CDDP doses (25 mg/m^2^)	Number of GEM doses (1,000 mg/m^2^)	Number of GEM doses (800 mg/m^2^)	Treatment duration (days)	Cause of discontinuation
1	1	71	Female	2	1	1	63	Second dose reduction
2	1	66	Male	14	3	11	172	Completion of protocol treatment
3	1	58	Male	13	13	0	162	Completion of protocol treatment
4	1	70	Male	13	8	5	169	Recurrence of tumor
5	1	71	Female	9	6	3	136	Second dose reduction
6	1	55	Male	7	4	3	115	Second dose reduction
7	1	72	Male	10	10	0	119	Second dose reduction
8	1	74	Male	4	1	3	51	Second dose reduction
9	1	61	Male	7	4	3	84	Second dose reduction
10	1	71	Male	9	9	0	149	Not satisfy starting criteria for creatinine
11	1	68	Male	16	9	7	176	Completion of protocol treatment
12	1	72	Female	10	9	1	113	Second dose reduction
13	1	71	Male	12	12	0	160	Completion of protocol treatment
14	1	73	Female	15	15	0	169	Completion of protocol treatment
15	1	66	Male	5	3	2	79	Second dose reduction
16	1	64	Female	1	1	0	15	Withdrew consent
17	1	64	Male	14	9	5	169	Not satisfy starting criteria for creatinine
18	1	73	Male	15	15	0	183	Completion of protocol treatment

## Discussion

Looking to improve the outcome of patients with BTC, we conducted a Phase I study of GC as adjuvant chemotherapy for BTC in patients undergoing curative resection without major hepatectomy. To the best of our knowledge, this is the first study which investigates the safety and feasibility of standard GC regimen as adjuvant setting for BTC.

Gemcitabine is rapidly metabolized to an inactive metabolite, 2′,2′-difluorodeoxyuridine by cytidine deaminase [15], which is expressed at varying levels in the human tissues [16], and the rapid clearance of gemcitabine can be attributed to its plentiful occurrence in the liver [17]. We were worried that a severe adverse event during adjuvant chemotherapy might occur after major hepatectomy and that patients would not tolerate standard GC therapy. Therefore, we excluded patients with BTC undergoing major hepatectomy on the basis of findings that patients with elevated bilirubin levels are at increased risk of hepatic injury with gemcitabine treatment [18] and that partially hepatectomized rats showed an increase in the area under the curve of cisplatin [19]. For the patients with BTC undergoing major hepatectomy, we are currently conducting another Phase I trials which is expected to define the RD of gemcitabine or S-1, respectively.

With regard to the treatment of advanced BTC, the BT-22 trial in Japan [14] showed a higher frequency of hematological adverse events during GC treatment than the ABC-02 trial in the UK [13]. Particularly with the GC regimen, rates of grade 3–4 neutropenia, leucopenia, thrombocytopenia and anemia, respectively, were 56.1, 29.3, 39.0 and 36.6 % in the BT-22 trial versus 25.3, 15.7 8.6, and 7.6 % in the ABC-02 trial. These differences in the frequency of hematological adverse events might have been due to ethnic differences. In this present adjuvant study, grade 3–4 neutropenia and leucopenia were more common with rates of 56 and 33 % of cases during the first 6 weeks and 94 % and 56 % throughout the entire course of treatment. We experienced more frequent hematological adverse events compared to the BT-22 trial; however, we speculate that postoperative damage might be related to such adverse events. Despite these relatively severe hematological adverse events, only one patient suffered from pneumonia with grade 1–2 neutropenia, and all adverse events were manageable.

Although hematological adverse events with the standard dose of GC were more frequent in the adjuvant than in the advanced setting, the standard dose might nevertheless be feasible for adjuvant chemotherapy for BTC in patients undergoing curative resection without major hepatectomy. From this study, we have defined the standard dose of GC as the RD under careful observation. Because our present protocol required that patients needing a second dose reduction should be off protocol, eight of 18 patients (44 %) resulted in cessation of protocol treatment due to second dose reduction. However, now we are planning to start a subsequent phase II trial for patients in this setting, and the criteria for dose reduction should be revised to allow well-planned second dose reduction, which would in turn allow more intensive cumulative dosing with adjuvant GC. Furthermore, we have to pay more attention to patient care to prevent severe adverse events, and further clinical trials will be warranted to improve the outcome of patients with BTC.
